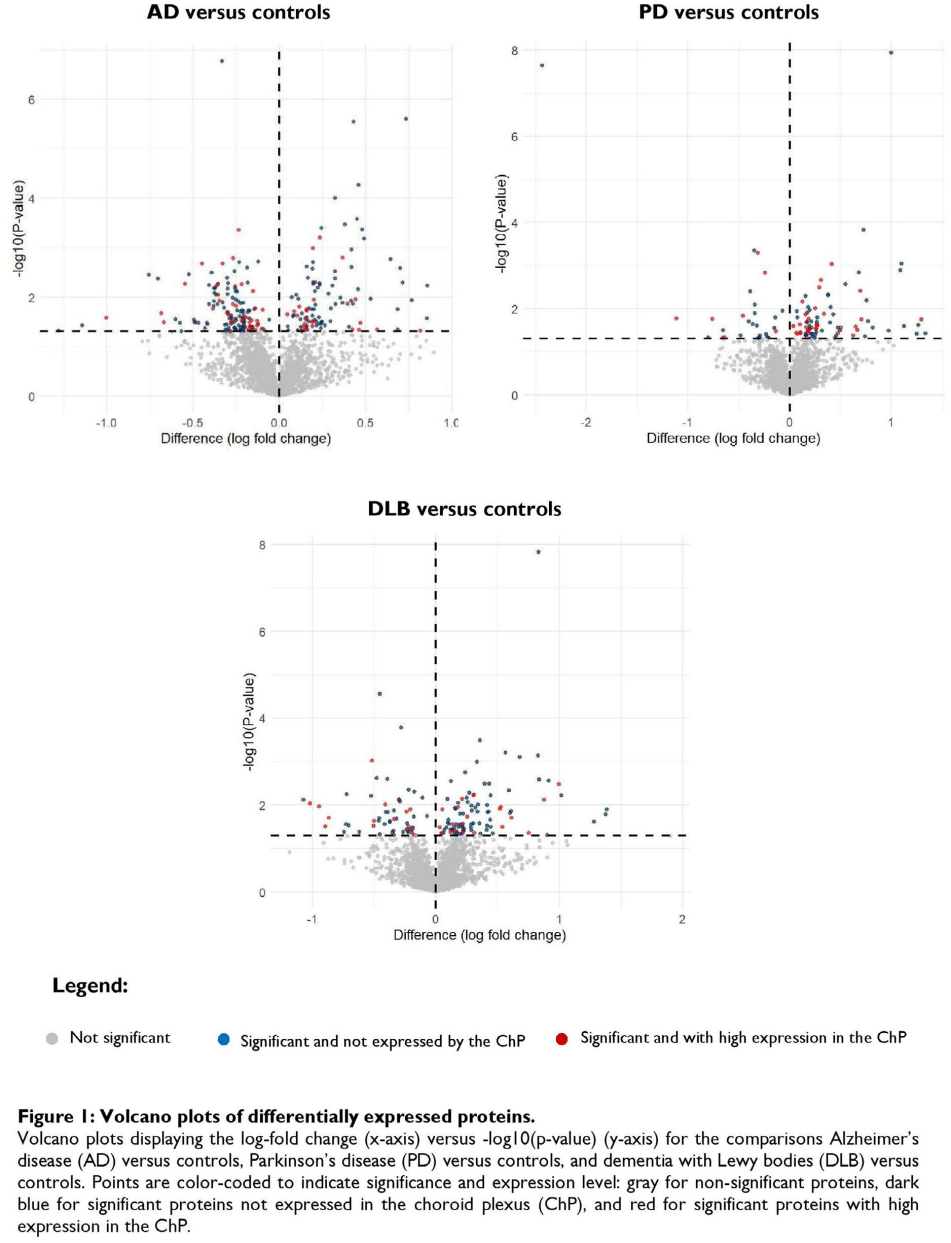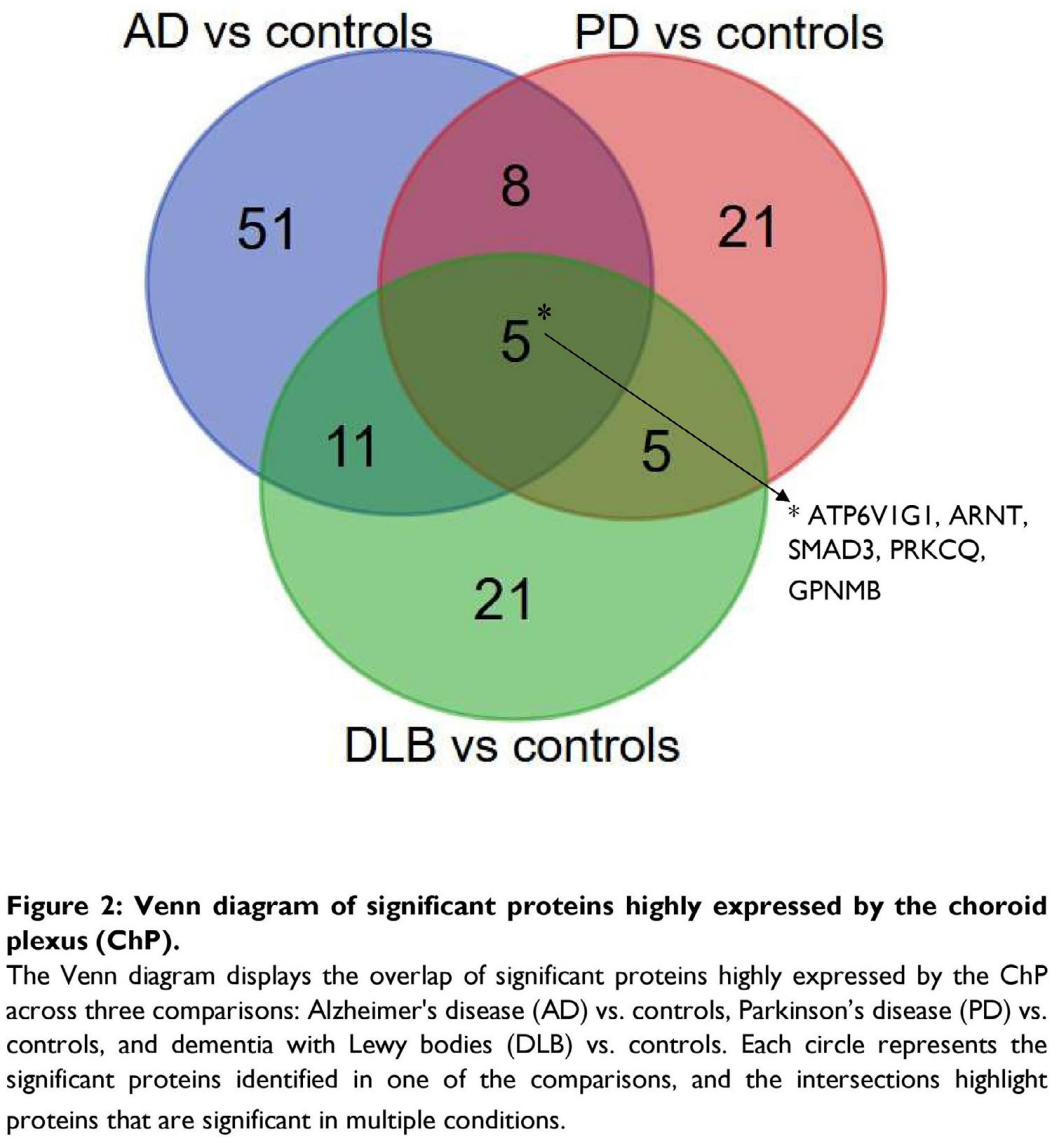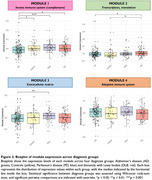# CSF proteomics reveals distinct choroid plexus‐related protein signatures in AD, PD and DLB

**DOI:** 10.1002/alz70855_102631

**Published:** 2025-12-23

**Authors:** Aurore Delvenne, Marianna Rizzo, Bailin Zhang, Mikhail Levit, Henrik Zetterberg, Kaj Blennow, Gwendlyn Kollmorgen, Rejko Krüger, Sara B. Gomes Fernandes, Dag Aarsland, Olga Borejko, Davit Chokoshvili, Wiesje M. van der Flier, Afina W. Lemstra, Betty M. Tijms, Gabor C Petzold, Annika Spottke, Giovanni B. Frisoni, Kathrin Brockmann, Thomas Gasser, Tormod Fladby, Marianne Wettergreen, Frank Jessen, Emrah Düzel, Günter U Höglinger, Claire Chevalier, Rajaraman Krishnan, Charlotte E. Teunissen, Stephanie J. B. Vos, Pieter Jelle Visser, IMI EPND consortium

**Affiliations:** ^1^ Alzheimer Center Limburg, School for Mental Health and Neuroscience, Maastricht University, Maastricht, Netherlands; ^2^ Sanofi, Cambridge, MA, USA; ^3^ Hong Kong Center for Neurodegenerative Diseases, Hong Kong, Science Park, China; ^4^ Clinical Neurochemistry Laboratory, Sahlgrenska University Hospital, Mölndal, Västra Götalands län, Sweden; ^5^ Wisconsin Alzheimer's Disease Research Center, University of Wisconsin‐Madison, School of Medicine and Public Health, Madison, WI, USA; ^6^ Department of Neurodegenerative Disease, UCL Institute of Neurology, Queen Square, London, United Kingdom; ^7^ UK Dementia Research Institute, University College London, London, United Kingdom; ^8^ Department of Psychiatry and Neurochemistry, Institute of Neuroscience and Physiology, University of Gothenburg, Mölndal, Sweden; ^9^ Clinical Neurochemistry Laboratory, Sahlgrenska University Hospital, Mölndal, Sweden; ^10^ Roche Diagnostics GmbH, Penzberg, Germany; ^11^ Transversal Translational Medicine, Luxembourg Institute of Health, Strassen, Luxembourg; ^12^ Luxembourg Centre for Systems Biomedicine, University of Luxembourg, Esch‐sur‐Alzette, Luxembourg; ^13^ Parkinson Research Clinic, Centre Hospitalier de Luxembourg, Luxembourg, Luxembourg; ^14^ Centre for Age‐Related Medicine, Stavanger University Hospital, Stavanger, Stavanger, Norway; ^15^ Institute of Psychiatry, Psychology & Neuroscience, King's College London, London, United Kingdom; ^16^ Institute of Psychiatry, Psychology and Neuroscience, King's College London, London, United Kingdom; ^17^ Luxembourg National Data Service, Esch‐sur‐Alzette, Luxembourg; ^18^ Alzheimer Center, Department of Neurology, Amsterdam UMC, Vrije Universiteit Amsterdam, Amsterdam Neuroscience, Amsterdam, Netherlands; ^19^ Department of Epidemiology and Data Science, Vrije Universiteit Amsterdam, Amsterdam UMC, Amsterdam, North Holland, Netherlands; ^20^ Alzheimer Center Amsterdam, Neurology, Vrije Universiteit Amsterdam, Amsterdam UMC location VUmc, Amsterdam, Netherlands; ^21^ Amsterdam Neuroscience, Neurodegeneration, Amsterdam, North Holland, Netherlands; ^22^ Alzheimer Center Amsterdam, Department of Neurology, Amsterdam Neuroscience, Vrije Universiteit Amsterdam, Amsterdam UMC, Amsterdam, Netherlands; ^23^ German Center for Neurodegenerative Diseases (DZNE), Bonn, NRW, Germany; ^24^ Division of Vascular Neurology, Department of Neurology, University Hospital Bonn, Bonn, NRW, Germany; ^25^ Department of Neurology, University of Bonn, Bonn, Germany; ^26^ German Center for Neurodegenerative Diseases (DZNE), Venusberg‐Campus 1, 53127, Bonn, Germany; ^27^ Memory Clinic, Geneva University Hospitals, Geneva, Switzerland; ^28^ Laboratory of Neuroimaging of Aging, University of Geneva, Geneva, Switzerland; ^29^ Hertie Institute for Clinical Brain Research, Department of Neurodegenerative Diseases, University of Tübingen, Tübingen, Germany; ^30^ German Center for Neurodegenerative Diseases (DZNE), Tübingen, Germany; ^31^ Department of Neurology, Akershus University Hospital, Lørenskog, Norway; ^32^ Institute of Clinical Medicine, University of Oslo, Oslo, Norway; ^33^ German Center for Neurodegenerative Diseases (DZNE), Bonn, Germany; ^34^ Department of Psychiatry, University of Cologne, Medical Faculty, Kerpener Strasse 62, Cologne, Germany; ^35^ Excellence Cluster on Cellular Stress Responses in Aging‐Associated Diseases (CECAD), University of Cologne, Cologne, Germany; ^36^ German Center for Neurodegenerative Diseases (DZNE), Magdeburg, Germany; ^37^ Institute of Cognitive Neurology and Dementia Research (IKND), Otto‐von‐Guericke University, Magdeburg, Sachsen Anhalt, Germany; ^38^ Department of Neurology, Klinikum der Ludwig‐Maximilians Universität München, Munich, Germany; ^39^ German Center for Neurodegenerative Diseases (DZNE), Munich, Germany; ^40^ Neurochemistry Laboratory, Department of Laboratory Medicine, Amsterdam Neuroscience, Vrije Universiteit Amsterdam, Amsterdam UMC, Amsterdam, Netherlands; ^41^ Alzheimer Center Limburg, Mental Health and Neuroscience Research Institute, Maastricht University, Maastricht, Netherlands; ^42^ Alzheimer Center Amsterdam, Department of Neurology, Vrije Universiteit Amsterdam, Amsterdam UMC location VUmc, Amsterdam, Netherlands; ^43^ Department of Neurobiology, Care Sciences and Society, Division of Neurogeriatrics, Karolinska Institutet, Stockholm, Sweden

## Abstract

**Background:**

The choroid plexus (ChP) plays a role in cerebrospinal fluid (CSF) production, protein transport and clearance, and central nervous system homeostasis. Emerging evidence suggests that ChP dysfunction is implicated in the pathogenesis of neurodegenerative diseases. We aim to investigate ChP involvement in the pathophysiology of Alzheimer's disease (AD), Parkinson's disease (PD), and dementia with Lewy bodies (DLB) using CSF proteomics.

**Method:**

We included individuals with AD (*n* = 150), PD (*n* = 75), DLB (*n* = 53), and controls (*n* = 47) from 6 centers of the European Platform for Neurodegenerative Diseases (EPND) project. AD was defined as abnormal CSF Aβ42/40 (Roche NeuroToolKit) without meeting clinical criteria of PD or DLB. Controls had normal cognition and CSF Aβ42/40. Using the Olink Explore 3072 assay, 2902 CSF proteins were quantified. Proteins were classified as highly expressed in the ChP using the Allen Brain Atlas. Pairwise comparisons of protein concentrations between disease and controls were conducted, adjusting for age and sex. Weighted Gene Co‐expression Network Analysis (WGCNA) was performed on ChP‐expressed proteins to identify co‐expression modules, followed by Wilcoxon tests to compare module expression profiles across groups. Pathway enrichment analysis was conducted using Gene Ontology.

**Result:**

Of the 2902 proteins quantified, 799 were highly expressed by the ChP. In PD versus controls, 37% of the dysregulated proteins were highly expressed in the ChP, compared to 30% in AD versus controls and 28% in DLB versus controls (Figure 1). The dysregulated ChP‐expressed proteins in AD, PD and DLB were predominantly distinct and associated with different underlying pathways (Figure 2). WGCNA identified four co‐expression modules related to the proteins with high ChP expression. Module 1, related to innate immunity (complement), was decreased in AD versus all other groups. Module 2, linked to transcriptional and translational processes, was increased in PD versus controls and AD. Module 3, related to the extracellular matrix, was increased in DLB versus AD. No significant differences were observed for module 4 (Figure 3).

**Conclusion:**

Our findings highlight the distinct involvement of ChP‐expressed proteins in the pathophysiology of AD, PD, and DLB, revealing disease‐specific patterns. Further research is essential to elucidate mechanistic involvement of the ChP in disease progression across these disorders.